# Supplementary material for: Azotobacter vinelandii glutaredoxin D delivers the core [Fe2S2] cluster to nitrogenase cofactor scaffold protein NifU
Source: J Biol Chem. 2026 Jul 16;302(8):113261. doi: 10.1016/j.jbc.2026.113261 (PMC13382767; doi:10.1016/j.jbc.2026.113261)
Supplement: Supplementary Material [file mmc1.pdf]

Figure S1. Mutation in *grxD* does not affect non-diazotrophic growth under low iron conditions. Growth under non-diazotrophic, low iron conditions of wild type *A. vinelandii* strain (DJ), *grxD* in-frame mutant (DJ3045), and DJ3045 transformed with a wild-type copy of *grxD* (DC47). Bars represent the average ± SD (n=3).

Figure S2. Full length immunoblots shown in Figure 2.

Figure S3. $_{H}$NifU does not bind to the Strep-column. Immunodetection with an anti-NifU antibody of His-tagged NifU in flowthough (FT), washes (W1, W6, and W7), and elution (E1 and E2) fractions after passing through a Strep-column. Images show a representative assay (n=3).

Figure S4. GrxD and NifU proteins interacts independently of their tags. A. Immunodetection with an anti-His antibody of His-tagged GrxD in flowthrough (FT), washes (W1, W6, and W7), and elution (E1 and E2) fractions after incubation with Strep-tagged NifU and passed through a Strep-column. B. Same fractions as above but visualized with an anti-Strep antibody. C. Immunodetection with an anti-His antibody of His-tagged GrxD in flowthrough (FT), washes (W1, W6, and W7), and elution (E1 and E2) fractions after passing through a Strep-column. Images show a representative assay (n=3).

Figure S5. GrxD$_S$ protein interacts with $_{H}$NifU is size exclusion chromatography assays. A. Immunodetection with an anti-NifU antibody of His-tagged NifU ($_{H}$NifU) in elutions (E1-E9) fractions obtained after passing pure isolated $_{H}$NifU through a size exclusion column. B. Immunodetection with an anti-Strep antibody of Strep-tagged GrxD (GrxD$_S$) in elutions (E1-E9) fractions obtained after passing pure isolated GrxD$_S$ through a size exclusion column. C. Immunodetection with an anti-NifU antibody of $_{H}$NifU in elutions (E1-E9) fractions obtained after passing an equimolar $_{H}$NifU and GrxD$_S$ mix through a size exclusion column. D. Immunodetection with an anti-Strep antibody of GrxD$_S$ in the elutions (E1-E9) fractions used in panel C and obtained after passing an equimolar $_{H}$NifU and GrxD$_S$ mix through a size exclusion column.

Figure S6. Full length immunoblots shown in Figure 3.

Figure S7. ΔN and ΔC ${}_{H}$NifU do not bind to a Strep-column. A. Immunodetection with an anti-His antibody of His-tagged ΔN-NifU in flowthrough (FT), washes (W1, W6, and W7), and elution (E1-E4) fractions after passing through a Strep-column. B. Immunodetection with an anti-His antibody of His-tagged ΔC-NifU in flowthrough (FT), washes (W1, W6, and W7), and elution (E1-E4) fractions after passing through a Strep-column. Images show a representative assay (n=3).

Figure S8. GrxD$_{S}$ does not transfer iron to ${}_{H}$NifU. Iron content of ${}_{H}$NifU (containing the core [Fe_2S_2] group), GrxD$_{S}$ (also purified with [Fe_2S_2] clusters), and ${}_{H}$NifU after being incubated and then separated from GrxD$_{S}$. Bars represent the average ± SD (n=3).

Figure S9. Apo-CA-${}_{H}$NifU receives a [Fe_2S_2] cluster from GrxD$_{S}$. A) Iron content of GrxD$_{S}$ and apo-CA-${}_{H}$NifU prior to interaction, and in the flowthrough fraction (FT CA-${}_{H}$NifU) obtained from passing through a Strep-tactin-column a solution in which apo-CA-${}_{H}$NifU was incubated for 15 min with GrxD$_{S}$. Bars represent the average ± SD (n=3). * Indicates statistically significant difference (p <0.01). B) Immunoblots of flowthrough (FT), wash (W1-W7) and elution (E) fraction of the previous solution performed with anti-Strep and anti-NifU antibodies to confirm that not GrxD$_{S}$ contamination was detected in the FT fraction. Images show a representative assay (n=3).

Figure S10. Full length immunoblots shown in Figure 4.

Figure S11. Apo-CA-${}_{H}$NifU does not receive a [Fe_2S_2] cluster from IscU$_{S}$. Iron content of IscU$_{S}$ and apo-CA-${}_{H}$NifU prior to interaction, and in the flowthrough fraction (FT CA-${}_{H}$NifU) obtained from passing through a Strep-tactin-column a solution in which apo-CA-${}_{H}$NifU was incubated for 15 min with IscU$_{S}$. Bars represent the average ± SD (n=3).

Figure S12. R-NifU$_{S}$ does not transfer clusters to ${}_{H}$GrxD. Iron content in reconstituted NifU (R-NifU$_{S}$), apo-${}_{H}$GrxD, and apo-${}_{H}$GrxD after being incubated with R-NifU$_{S}$ and separated with a Strep-tactin column. Bars represent the average ± SD (n=3).

Figure S13. Apo-CA-${}_{H}$NifU receives a [Fe_2S_2] cluster from GrxD$_{S}$ independent of reducing agent used. Iron content of GrxD$_{S}$ and apo-CA-${}_{H}$NifU prior to interaction, and in the flowthrough fraction (FT CA-${}_{H}$NifU) obtained from passing through a Strep-tactin-

column a solution in which apo-CA-$_H$NifU was incubated for 15 min with GrxD$_S$ in the presence of 5 mM GSH instead of DTT. Bars represent the average ± SD (n=3). * Indicates statistically significant difference (*p* <0.01).

Figure S14. Temperature dependent relaxation of X-EPR spectra. A. X-band CW-EPR spectra of CA-$_H$NifU measured at different temperatures indicated in the figure. The signal intensity and line width are identical at 15 K and 50 K, suggesting that the S = ½ species is most likely a $[2Fe2S]^+$ cluster. B. X-band CW-EPR spectra of CA-$_H$NifU (A), GrxD$_S$ (B), flowthrough fraction after 15 min interaction of apo-CA-$_H$NifU and GrxD$_S$ (C) apo-CA-$_H$NifU (D) measured at 15 K. For B and D, the signals were multiplied by 20 times. A single S = ½ species with g = [2.02, 1.93, 1.89] is observed. The spin concentration of this species is 0.5 mM (A), 0 mM (B), 0.73 mM (C), and <10 µM (D).

Figure S15. Reconstituted $_H$NifU for NifH reconstitution assays in Fig. 5 does not carry over detectable GrxD$_S$. A) $_H$NifU is separated of GrxD$_S$ by Strep-tactin column and immunodetected with anti-Strep antibody in flowthrough (FT), washes (W) 1 and 7, and in elution (E) fractions. B) Coomassie blue stained gel corresponding to the immunoblot in A). Images show a representative assay (n=3).

Figure 16. GrxD is required for optimal NifU iron occupancy *in vivo*. A) Coomassie Blue Stain gel from NifU$_S$ obtained from *A. vinelandii* strains DC216 (control strain expressing NifU$_S$ under its own promoter in a DJ background), DC214 (expressing NifU$_S$ under its own promoter in a *ΔgrxD* background), DC215 (producing CA-NifU$_S$ in DJ), and DC219 (producing CA-NifU$_S$ in *ΔgrxD*). Dobrzyńska *et al* (2024) identified the three main co-purified proteins as biotin-containing pyruvate carboxylase (bands 1 and 2), and acetyl-coA carboxylase (band 3) (50); proteins that do not use iron cofactors. B) Iron content of NifU$_S$ obtained from *A. vinelandii* DC216, DC 214, DC215, and DC219 (n =2). NifU protein levels were calculated using a reference dilution series of pure NifU$_S$ obtained from *E. coli.* * Indicates statistically significant difference (*p* <0.01). C. UV-vis spectra normalized with protein absorbance (OD_{280}) from DC216 (orange), DC214 (green), DC219 (blue) and DC215 (black).
