# Supplementary material for: Azotobacter vinelandii glutaredoxin D delivers the core [Fe2S2] cluster to nitrogenase cofactor scaffold protein NifU
Source: J Biol Chem. 2026 Jul 16;302(8):113261. doi: 10.1016/j.jbc.2026.113261 (PMC13382767; doi:10.1016/j.jbc.2026.113261)
Supplement: Supplementary Material [file mmc2.pdf]

**FIGURE S1**

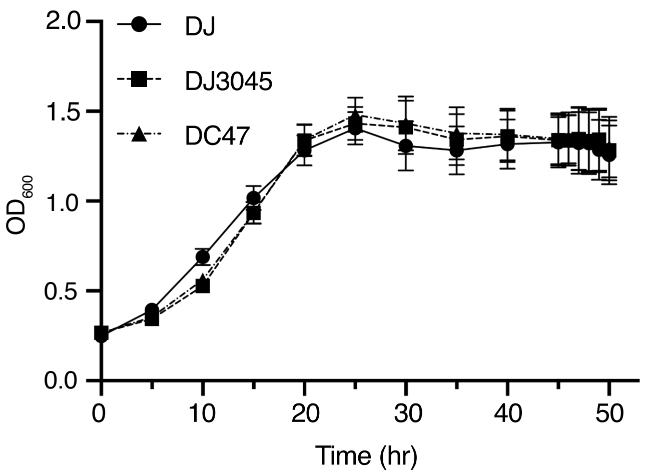

**FIGURE S2**

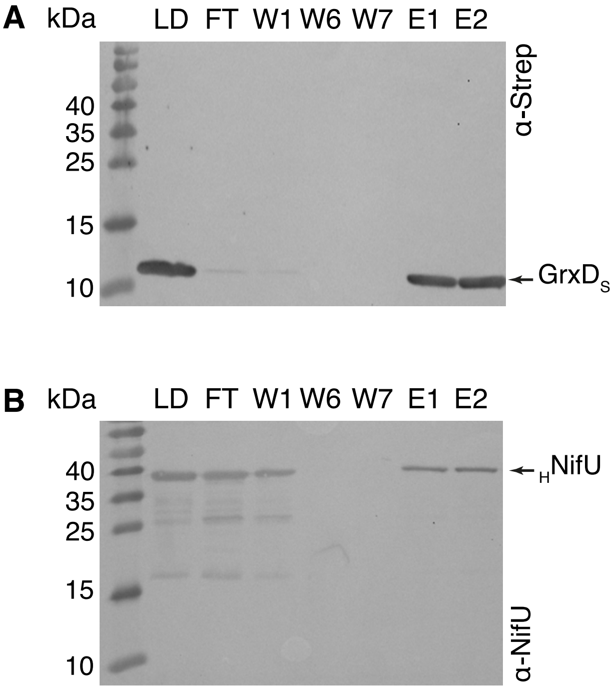
A
kDa
LD FT W1 W6 W7 E1 E2
40
35
25
15
10
α-Strep
GrxD$_S$
B
kDa
LD FT W1 W6 W7 E1 E2
40
35
25
15
10
$_H$NifU
α-NifU

**FIGURE S3**

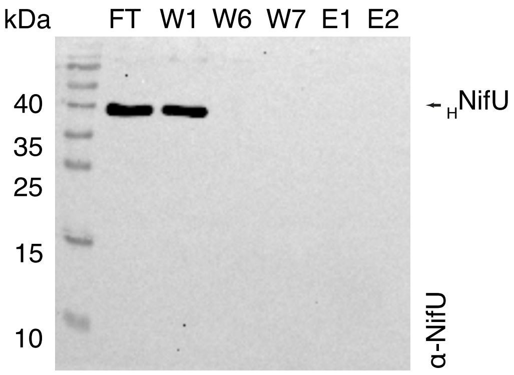

**FIGURE S4**

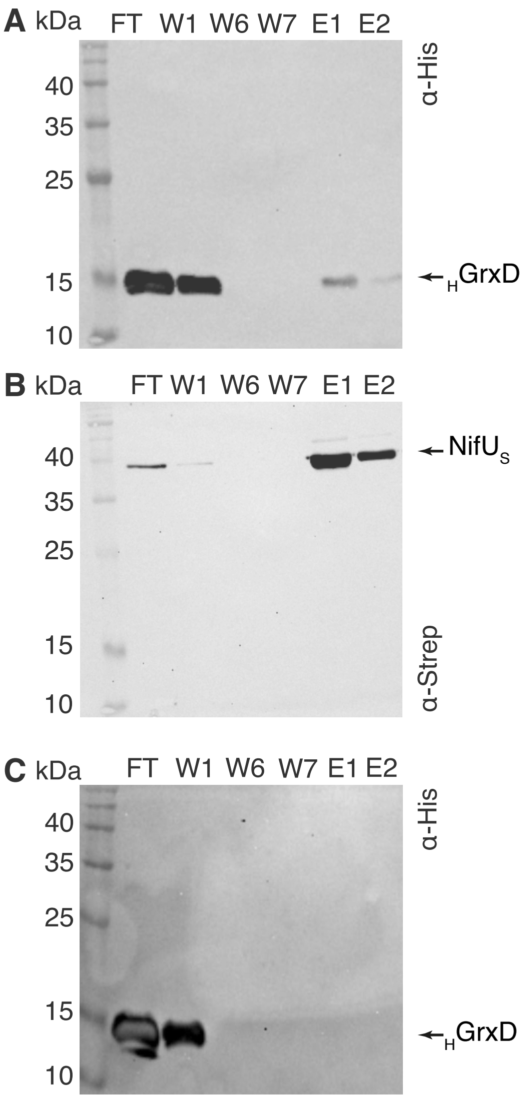

A kDa
FT W1 W6 W7 E1 E2
40
35
25
15
10
α-His
←HGrxD
B kDa
FT W1 W6 W7 E1 E2
40
35
25
15
10
←NifUS
α-Strep
C kDa
FT W1 W6 W7 E1 E2
40
35
25
15
10
α-His
←HGrxD

## FIGURE S5

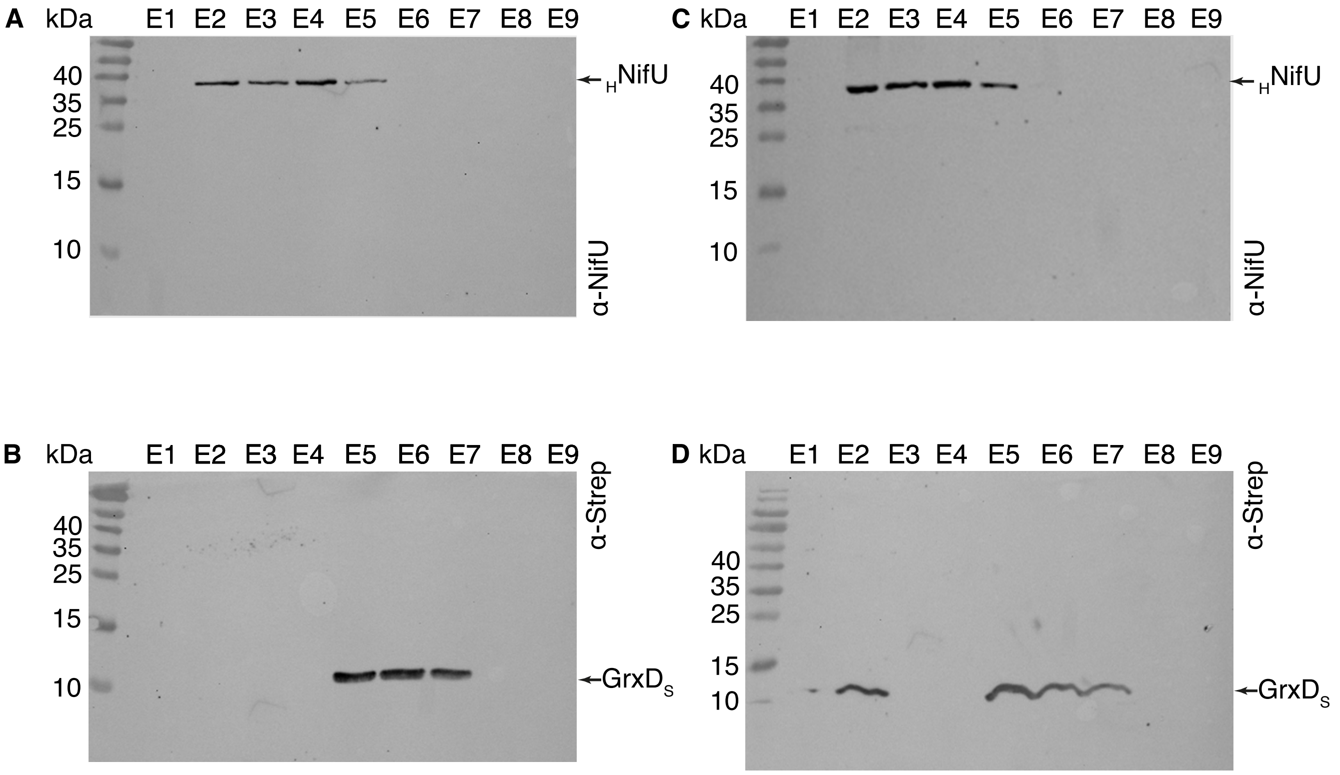
A kDa E1 E2 E3 E4 E5 E6 E7 E8 E9
40
35
25
15
10
HNifU
α-NifU
C kDa E1 E2 E3 E4 E5 E6 E7 E8 E9
40
35
25
15
10
HNifU
α-NifU
B kDa E1 E2 E3 E4 E5 E6 E7 E8 E9
40
35
25
15
10
α-Strep
GrxDS
D kDa E1 E2 E3 E4 E5 E6 E7 E8 E9
40
35
25
15
10
α-Strep
GrxDS

## FIGURE S6

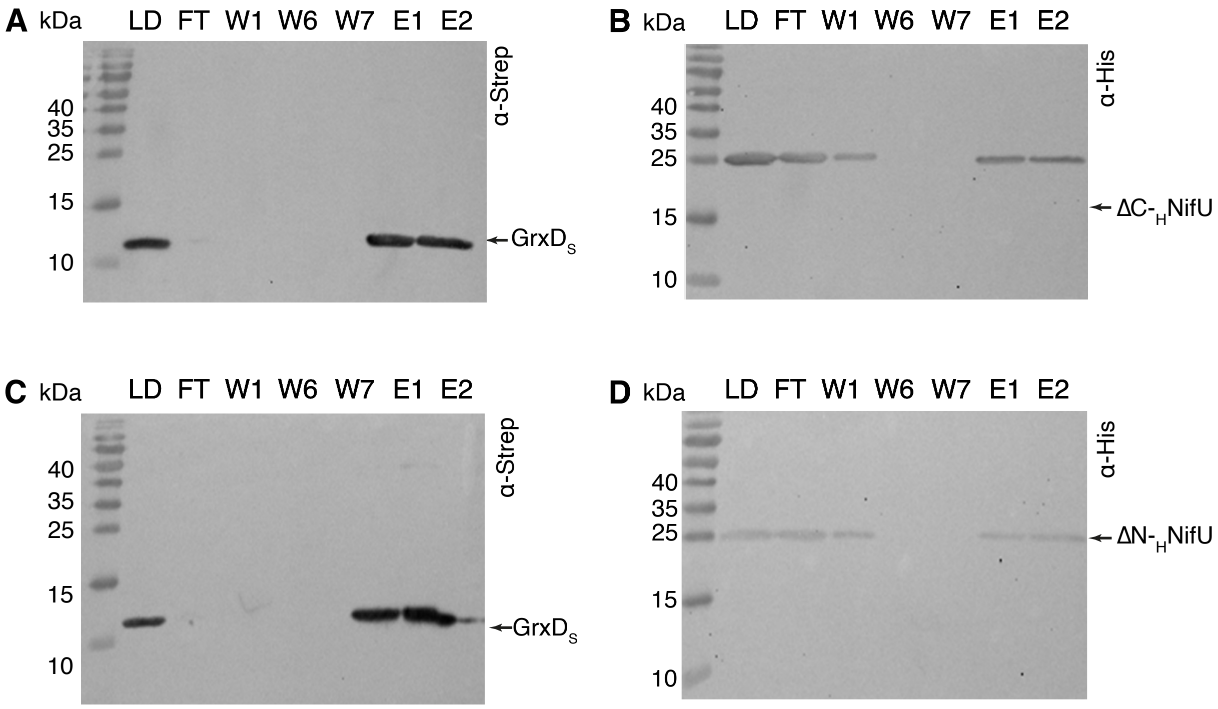

A kDa LD FT W1 W6 W7 E1 E2
40
35
25
15
10
α-Strep
GrxD$_S$
B kDa LD FT W1 W6 W7 E1 E2
40
35
25
15
10
α-His
ΔC-$_H$NifU
C kDa LD FT W1 W6 W7 E1 E2
40
35
25
15
10
α-Strep
GrxD$_S$
D kDa LD FT W1 W6 W7 E1 E2
40
35
25
15
10
α-His
ΔN-$_H$NifU

**FIGURA S7**

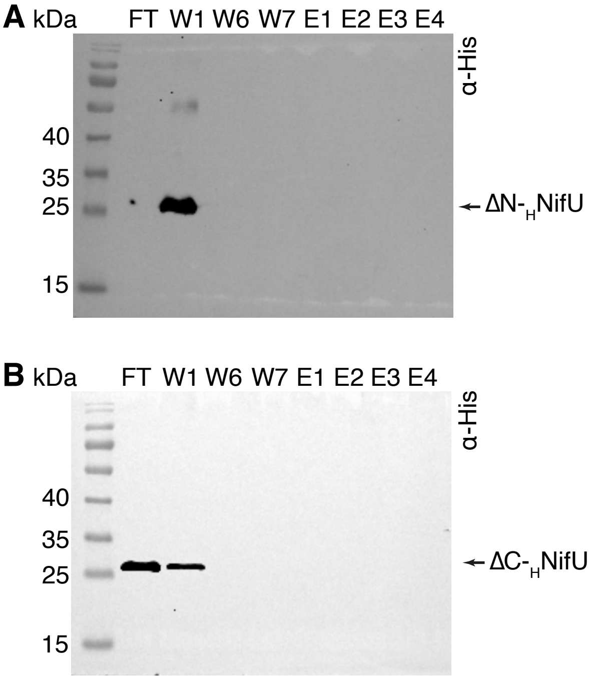
A kDa
FT W1 W6 W7 E1 E2 E3 E4
α-His
40
35
25
15
ΔN-HNifU
B kDa
FT W1 W6 W7 E1 E2 E3 E4
α-His
40
35
25
15
ΔC-HNifU

**FIGURE S8**

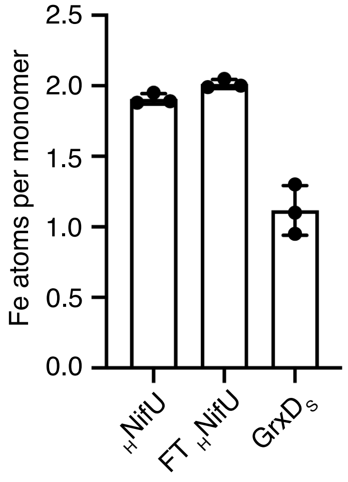

**FIGURE S9**

A

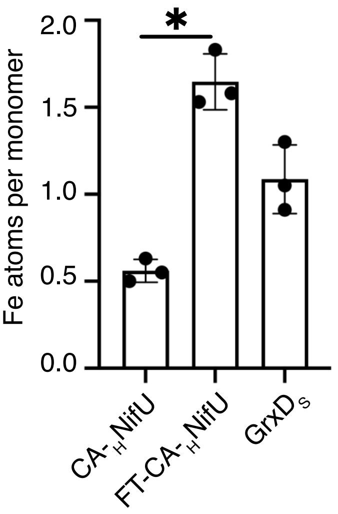
*
2.0
1.5
1.0
0.5
0.0
Fe atoms per monomer
CA-HNifU
FT-CA-HNifU
GrxDS

B

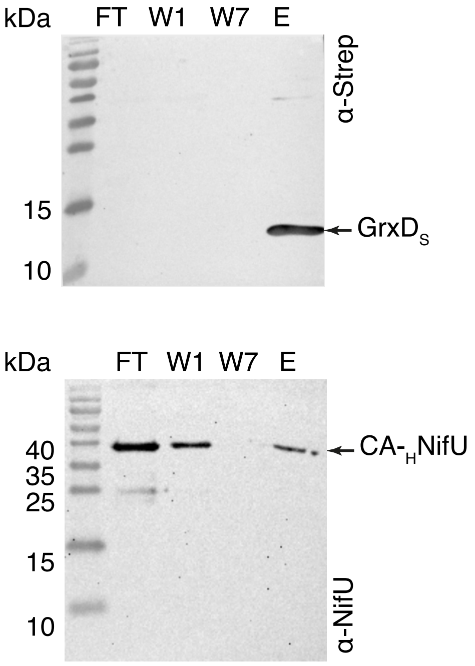
kDa
FT
W1
W7
E
α-Strep
15
10
GrxDS
kDa
FT
W1
W7
E
40
35
25
15
10
CA-HNifU
α-NifU

## FIGURE S10

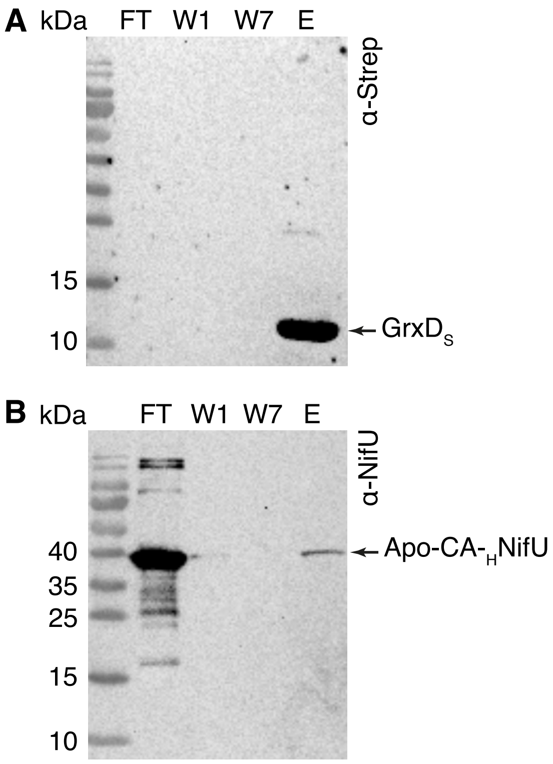
A
kDa
FT
W1
W7
E
α-Strep
15
10
GrxD$_S$
B
kDa
FT
W1
W7
E
α-NifU
40
35
25
15
10
Apo-CA-$_H$NifU

**FIGURE S11**

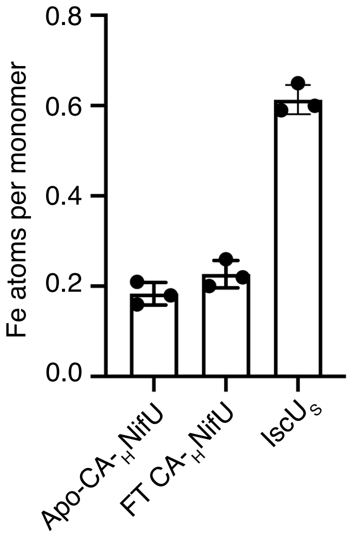

0.8
0.6
0.4
0.2
0.0
Fe atoms per monomer
Apo-CA-$_H$NifU
FT CA-$_H$NifU
IscU$_S$

**FIGURE S12**

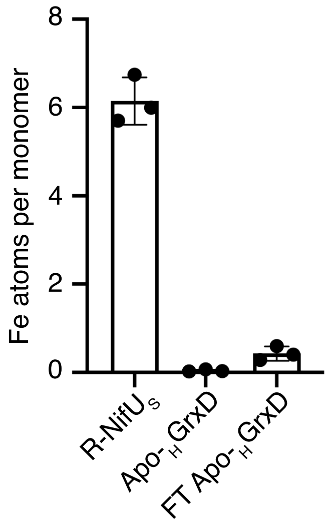

**FIGURE S13**

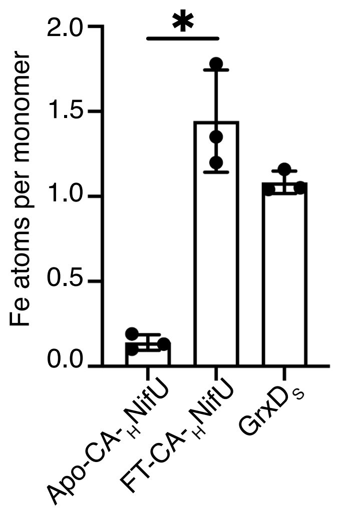

Fe atoms per monomer
2.0
1.5
1.0
0.5
0.0
*
Apo-CA-HNifU
FT-CA-HNifU
GrxDS

# FIGURE S14

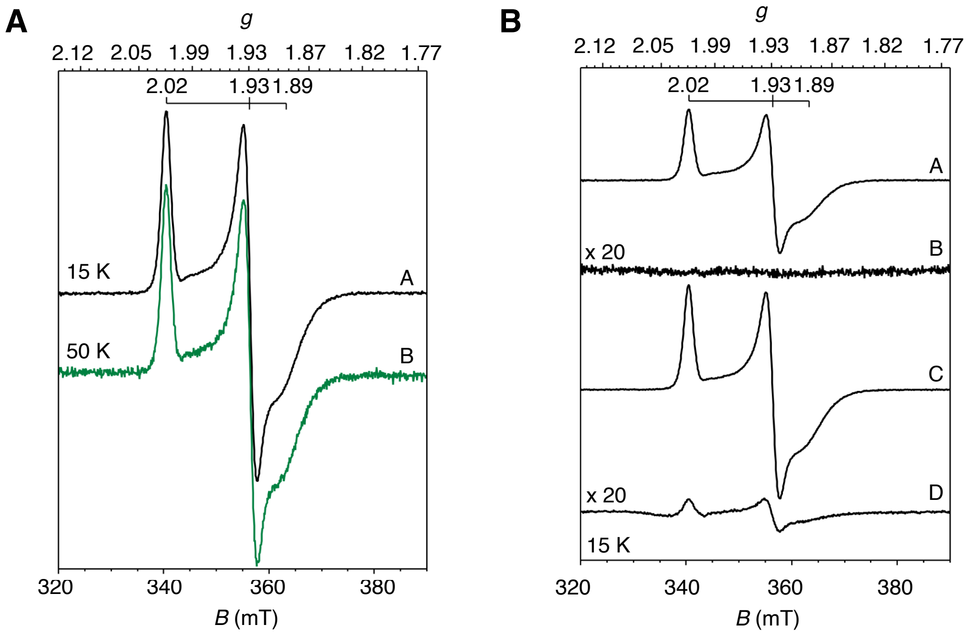

A
g
2.12
2.05
1.99
1.93
1.87
1.82
1.77
2.02
1.93
1.89
15 K
A
50 K
B
320
340
360
380
B (mT)
B
g
2.12
2.05
1.99
1.93
1.87
1.82
1.77
2.02
1.93
1.89
A
x 20
B
C
x 20
D
15 K
320
340
360
380
B (mT)

**FIGURE S15**

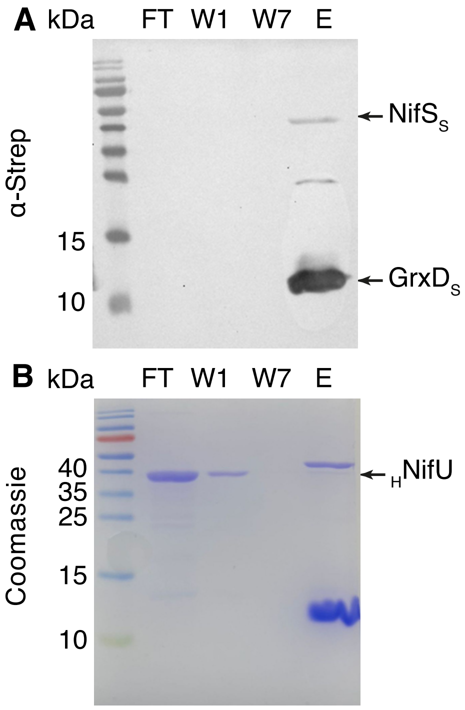
A kDa
FT W1 W7 E
α-Strep
15
10
NifS$_S$
GrxD$_S$
B kDa
FT W1 W7 E
Coomassie
40
35
25
15
10
$_H$NifU

**FIGURE S16**

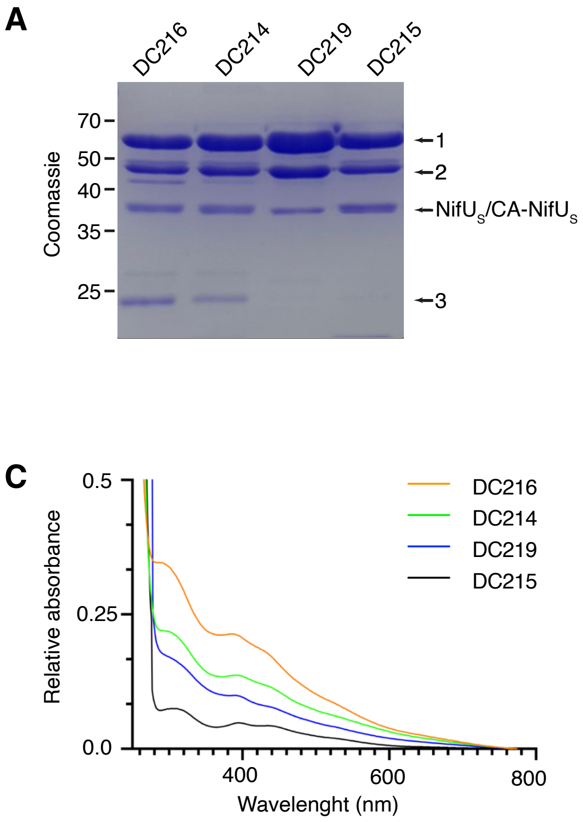
A
DC216
DC214
DC219
DC215
70
50
40
35
25
Coomassie
1
2
NifU$_S$/CA-NifU$_S$
3
B
*
*
Fe atoms per monomer
6
4
2
0
DC216
DC214
DC219
DC215
C
0.5
0.25
0.0
Relative absorbance
DC216
DC214
DC219
DC215
400
600
800
Wavelenght (nm)
