# Supplementary material for: Azotobacter vinelandii glutaredoxin D delivers the core [Fe2S2] cluster to nitrogenase cofactor scaffold protein NifU
Source: J Biol Chem. 2026 Jul 16;302(8):113261. doi: 10.1016/j.jbc.2026.113261 (PMC13382767; doi:10.1016/j.jbc.2026.113261)
Supplement: Supplementary Material [file mmc3.pdf]

Escherichia coli and Azotobacter vinelandii strains and plasmids

To produce IscU$_S$, IscU was amplified from *A. vinelandii* DNA using the primers 5*Nco*IIscU-C-Strep and 3*Nde*I-IscU-C-Strep (Table S1), digested with *Nco*I and *Nde*I restriction enzymes and cloned in pET16bStrep to produce pERN6 (Table S2).

A. vinelandii strains DC214 and DC216 were generated by transformation with vector pASC75 of *A. vinelandii* DJ3045 and DJ, respectively. Strains DC215 and DC219 were produced by transforming *A. vineladii* DJ3045 and DJ with vector pASC76, respectively. These vectors were obtained by amplifying *nifU* using primers Fwd-*nifU*-1, Rev-strep-tag-Ct, and Fwd-strep-tag-Ct and Rev-*nifS*-169 (Table S1) from pASC49 (which contains native *nifU*) or from pERN5 and placing a Strep-tag at the C-terminus of *nifU* after residue 303. PCR products were cloned into PciI-AscI sites of pASC74 (containing NifUS and flanking región from 1038 bp upstream to 1060 downstream). Transformation of *A. vinelandii* was carried out as described (42, 43). To select for the transformants, the strains were co-transformed with pDB303 (to select with 10 µg/mL Rifampicin for DC47, DC215, and DC219) and pDB1416 (to select with 0.05 µg/mL Gentamycin for DJ3045) provided by Dr. Dennis Dean (Virginia Tech, Blacksburg, USA).

Size exclusion chromatography to assess protein-protein interactions

$_H$NifU–GrxD$_S$ interactions were analyzed using an Agilent 1260 Infinity II LC (Agilent Technologies, Santa Clara, CA, USA) equipped with an AdvanceBio SEC 200 Å column (1.9 µm, 4.6 × 300 mm). Size-exclusion chromatography was performed at a flow rate of 0.35 ml min^{-1} using O_2-free, de-gassed 100 mM Tris pH 8.0, 150 mM NaCl buffer. Protein samples (injecting volume of 10 µl containing 1 nmol of each protein) were mixed and incubated at room temperature for 5 min prior to injection onto the column. Elution was monitored at 220 and 280 nm, and protein-containing fractions were collected at 1 min intervals from the total column volume (7 minutes, E1) until the last minute run (15 min, E9). Eluted protein fractions were loaded in SDS gels and, following electrophoresis, were transferred to 0.2 µm nitrocellulose membranes (Bio-Rad, Hercules, CA, USA). NifU and GrxD$_S$ were detected by Western blotting using anti-NifU and anti-Strep-MAB antibodies (IBA Lifesciences, Göttingen, Germany), respectively. Membrane images were collected using an iBright imaging apparatus (Thermo Fisher Scientific).

Protein purification from A. vinelandii cells

NifU$_S$ proteins produced in *A. vinelandi*i strains DC214, DC215, DC216, and DC219 were purified from a 150 l cultures grown in modified Burk's medium supplemented with ammonium acetate (5.5 mM) in a 300 l fermentor (Bioprocess Bioengineering) Nitrogenase de-repression was

initiated when cultures finished the nitrogen source around $OD_{600} = 2$. After 4 h of de-repression, cells were immediately collected and stored at −80 °C. 100 g of these cells were resuspended in the 100 ml buffer W supplemented with 1 mM PMSF and 3 mg DNAse I inside a CoyLabs glovebox (CoyLabs). Cells were lysed and the cell-free extract (CFE) was obtained after removing cell debris by centrifugation at 63,000 g for 1 h at 4 ºC. CFE was loaded onto a 5 ml flow Streptactin-XT column (IBA, Göttingen, Germany), previously equilibrated with buffer W. The column was then washed 5 times with five column-volumes (CV) of buffer W per wash. The bound proteins were eluted with 5 CV of 50 mM biotin in buffer W. Elution fractions were concentrated with centrifugal membrane devices (Amicon Ultra, Millipore, Burlington, MA, USA). The proteins were desalted using a was desalted by dilution and concentrated with a 10-kDa cutoff pore. To estimate the amount of NifU in the elutions, a calibration curve with pure $NifU_S$ was performed in a SDS-PAGE. Protein and iron content was determined using BCA (Pierce, IL, USA) and bipyridyl method (44), respectively.

[FeS] cluster transfer assays

To determine iron transfer between apo-CA-$_H$NifU with $IscU_S$, 50 µM of the NifU and 250 µM of IscU were incubated in a glovebox for 15 min at room temperature in buffer W. Proteins were separated by passing them through a column of 5 ml Strep-tactin XT 4Flow High-capacity column (IBA) previously equilibrated in buffer W. The column was washed with seven CVs of buffer W, and the proteins were eluted with 50 mM biotin in Buffer W.
