# Supplementary material for: Azotobacter vinelandii glutaredoxin D delivers the core [Fe2S2] cluster to nitrogenase cofactor scaffold protein NifU
Source: J Biol Chem. 2026 Jul 16;302(8):113261. doi: 10.1016/j.jbc.2026.113261 (PMC13382767; doi:10.1016/j.jbc.2026.113261)
Supplement: Supplementary Material [file mmc4.pdf]

Supplementary Table 1. Primers used in this study.

Use	Name	Sequence
$_{H}$GrxD cloning	5*Nde*IGdxN-is	AGGCATATGATGGATATCATCGAAACCATT
	3*Bam*HIGdxN-His	CCTGGATCCTCAAGCATCGGCTTTGTCGGC
GrxD$_{S}$ cloning	5*Nco*IGdxC-Strep	AGGCCATGGATATCATCGAAACCATTAAAG
	3*Nde*IGdxC-Strep	CCTCATATGAGCATCGGCTTTGTCGGCCGC
GrxD in frame mutant	FW-*Not*I-700UpGrx5	AAAGCGGCCGCCAGGGATGCAGATCGTCGGAC
	700DownGrx5*Eco*RI-RV	CCTGAATTCCAACCTCCAGGAGCTCGGCAA
IscU$_{S}$ cloning	FW-5*NcoI*-IscU-C-Strep	AGGCCATGGCTTACAGTGACAAGGTCATCG
	3*NdeI*-IscU-C-Strep	CCTCATATGAACCAGGCCTTTCTTGTGCTT
pASC75/pASC76 cloning	nifU_1 fwd	TGAAGCCGACATGTGGGATTATTCGGAAAA
	nifS 169 rev	GTGTGAAACATGATGCCGGC
	strep-tag Ct Fwd	GCTAGCTGGAGCCACCCGCAGTTCGAAAAGGCTGCGGCTCACGCGCAGATGGAGGTCTGA
	strep-tag Ct Rev	CTTTTCGAACTGCGGGTGGCTCCAGCTAGCGCTGACCGGAATCACCTTGACGAACTCGCC

Supplementary Table 2. Plasmid used in this work

Plasmid	Used for	Source
pRHB612	To overexpress 9x His- *nifU* under T7 promoter, Ap^R	Rubio laboratory collection
pDB303	This plasmid carries the Rif^R determinant	Dean laboratory collection
pDB1416	To interrupts *scrB* gene with Gentamycin cartridge	Dean laboratory collection
pDB2123	To overexpress streptag-*nifS* under T7 promoter, Ap^R.	Dean laboratory collection
pDB2174	To overexpress *nifU*-streptag under T7 promoter, Ap^R	Dean laboratory collection
pDB2678	To generate a in frame deletion mutant *grxD* aa2-50, Ap^R	Dean laboratory collection
pDB2679	To restore *grxD* gene in a *grxD* mutant, Ap^R	Dean laboratory collection
pERN1	To overexpress g*rxD*- strep tag under T7 promoter, Ap^R	This work
pERN2	To overexpress 9x His- g*rxD* under T7 promoter, Ap^R	This work
pERN3	To overexpress 9x His- ΔN - *nifU* under T7 promoter, Ap^R	This work
pERN4	To overexpress 9x His- ΔC- *nifU* under T7 promoter, Ap^R	This work
pERN5	To overexpress 9x His- CA- *nifU* under T7 promoter, Ap^R	This work
pERN6	To overexpress *IscU*- strep tag under T7 promoter, Ap^R	This work
pASC75	Puts a strep-tag at C-terminal of *nifU* after residue 303, Ap^R	This work
pASC76	Puts a strep-tag at C-terminal of *CA-nifU* after residue 303, Ap^R	This work
